# Progress in Surgery

**Published:** 1899-09-09

**Authors:** 


### Progress in Surgery.

### ORTHOPÆDIC SURGERY.

(*Continued from page* 385.)

**Hypertrophy of the Tibia.**—Dr. S. Ketch,[7] before the New York Academy of Medicine, presented a girl, four years of age, whose right tibia was greatly lengthened and thickened with decided anterior bowing. He had first seen the child in December, 1898. The epiphyses were thickened, and the enlargement was most marked at the middle of the shaft, but included the whole bone, as was seen by the X-rays. Length—Right leg, $19\frac{1}{2}$; left, $18\frac{5}{8}$; right tibia, $9\frac{1}{4}$; left, $8\frac{3}{4}$. Circumference—Right thigh, $9\frac{1}{2}$; left, $10\frac{1}{4}$; right calf, $8\frac{5}{8}$; left, $7\frac{7}{8}$. The disease had commenced 18 months before with a small lump on the leg and pain at night, and when she walked. This was his second patient of the kind. The first one was a girl, aged 11, who had been operated on for the purpose of shortening and straightening the bone, with resulting improvement and ability to walk about. The bones had been found to be solid, the cavity being obliterated. Neither of the patients had received any benefit from antisyphilitic treatment. It was possible that the trouble began in the periosteum. It was a question whether something ought to be done early to arrest the process, such as an incision through the periosteum, which might at least relieve the tension.

**Accidents in Orthopædic Surgery.**—Dr. Kaposi[8] describes a case in which an operation for flat foot was followed by serious symptoms almost resulting fatally. The patient was a boy, 15 years of age, in the surgical clinic of Heidelberg. An anæsthetic having been given the foot was supinated and fixed in that position by a plaster bandage. The father was told to bring the boy back in three weeks, but he reappeared in six days, complaining of great pain in the foot, which, on the removal of the bandage, was found to be swollen and inflamed. Wet compresses were therefore applied, and the boy was sent home at the father's request. In five days he returned with symptoms of septicæmia, having a temperature of 39·7 deg. C. (103·4 deg. Fahr.), and a pulse of 140. There was swelling and pain, especially over the cuboid and fifth metatarsal bones. A free incision was made, and more than a tablespoonful of pus was evacuated. The cuboid bone was partly necrosed, and a sequestrum was removed. After the operation the patient's state improved, but a fortnight later the fever recurred with swelling and pain over the ankle-joint. The pulse was exceedingly quick, and the boy's general state was worse than before. The joint was therefore incised and drained, after which the boy gradually recovered. The flat foot had, of course, become worse owing to the removal of nearly the whole of the cuboid bone. The case was obviously one of acute osteomyelitis following the reduction of the flat foot, apparently without any cause. Dr. Kaposi suggests a hæmatogenous infection. Another accident complicating treatment of club-foot was brought to his notice by Professor Jordan. The deformity was overcome by tenotomy of the tendo-Achillis and incision of the plantar fascia, and a plaster bandage was then applied. Severe pain followed, so that the plaster bandage had to be removed after a few days, but, as nothing abnormal was found, another bandage was applied.

The pains ceased, but marked paræsthesia, formication in the course of the internal plantar nerve, and itching ensued. The patient became sleepless and highly neurasthenic, and was unable to take food, so that she grew weak. The dressing was then entirely removed, and massage and electricity were employed. In time the pains were relieved, but they did not disappear in less than six months. Obviously an irritation of the plantar nerve had been caused by the stretching of the foot.

**Tendon Grafting for Drop-foot with Contracture of the Tendo-Achillis.**—Dr. V. P. Gibney[9] presented a case of a girl, aged 14, who had marked drop-foot with loss of power in the common extensor group of muscles. There was a fair degree of power in the anterior tibialis, but this was overcome by a shortened tendo-Achillis. The tendons on the anterior aspect of the ankle and foot were exposed, the tendon of the tibialis anticus was split about three inches, and the outer half sutured to the tendon of the common extensors. The tendo-Achillis was split nearly three inches, and its inner portion passed under the skin and sutured to the remainder of the tibialis anticus. The remainder of the tendo-Achillis was cut obliquely, and the tendon lengthened to the extent of about one and a half inches. The sutured end of the tibialis anticus and the tendo-Achillis was drawn over toward the common extensor, and all were sutured together with kangaroo tendon. After closure of the wounds the foot was put up in plaster-of-Paris. On the third or fourth day there was a slight rise of temperature; this continued, and at the end of the week the wound was exposed, when it was found that superficial sloughing had occurred. The parts were dressed daily until healing took place. The foot appeared to be normal; she could flex and extend it with a fair amount of force, and probably she would be able to dispense with all apparatus and have a very useful foot.

**Excision of Astragalus for Talipes Equino-Varus.**—Dr. Morton,[10] before the Philadelphia Academy of Surgery, presented several cases of club-foot from whom he had excised the astragalus for congenital club-foot of the equino-varus variety. The question had been raised whether in time ankylosis of the joint would result from this procedure. He stated that, after a large experience, in no instance had such ankylosis occurred, but good ankle motion had been secured. He recognised in equino-varus two forms, one the uncomplicated, the other the complicated club-foot; the former is corrected by tenotomy, stretching, massage, and apparatus; the latter is always associated with displacement, partial or complete, of the astragalus. This bone has been invariably forced forwards and out of its normal position; indeed, in some instances, the tibia is found articulating with the os calcis. The astragalus will in such a case be found quite subcutaneous and in advance of the tibia. While it remains in that position it prevents flexion of the foot; it is an obstacle to the foot being brought into a proper normal position. It, furthermore, becomes so changed in form that it could not perform its normal function in the articulation, even if it could be restored to its usual position. In operating for excision of the astragalus it is important to first perform very general tenotomy. This consists in dividing the flexor tendon of each toe, the tendo-Achillis, and the tendon of the anterior tibial (if found contracted). An incision should be made in a straight or curved line from the base of the fourth toe, over the most prominent portion of the astragalus to the external malleolus. Then the knife is readily carried around the circumference of the bone and all attachments are divided, and it can be removed with a curved scissors and a bone forceps. If there still remain any difficulty in placing the foot in a normal, right-angle position the cause will be found in the cuboid, or other tarsal bone, and the bone in whole or part should be removed.

[7] Med. Times and Register, June, 1899. [8] Lancet, July 8, 1899, p. 125.
[9] New York Med. Rec., Mar. 25, 1899. [10] Annals of Surgery, May, 1899.

